# Autologous Hematopoietic Cell Transplantation in Multiple Sclerosis: Changing Paradigms in the Era of Novel Agents

**DOI:** 10.1155/2019/5840286

**Published:** 2019-06-24

**Authors:** Maria Gavriilaki, Ioanna Sakellari, Eleni Gavriilaki, Vasilios K. Kimiskidis, Achilles Anagnostopoulos

**Affiliations:** ^1^Postgraduate Course, School of Medicine, Aristotle University of Thessaloniki, Thessaloniki, Greece; ^2^Hematology Department-BMT Unit, G. Papanicolaou Hospital, Thessaloniki, Greece; ^3^Laboratory of Clinical Neurophysiology, AHEPA Hospital, Aristotle University of Thessaloniki, Thessaloniki, Greece

## Abstract

Autologous hematopoietic stem cell transplantation (AHSCT) is established as a standard of care for diseases ranging from hematological malignancies to other neoplastic pathologies and severe immunological deficiencies. In April 1995, our group performed the first AHSCT in progressive multiple sclerosis (MS). Since then, a plethora of studies have been published with encouraging but controversial results. Major challenges in the field include appropriate patient selection, improvements in AHSCT procedure, and timing of this treatment modality. Beyond AHSCT, several new intravenous or oral agents have been developed and approved over the last 20 years in MS. The emergence of multiple effective therapies for MS has created a challenging scenario for both treating physicians and patients. Novel cell-based therapies other than AHSCT are also currently investigated in MS patients with promising results. Our review is aimed at summarizing state-of-the-art knowledge on basic principles and results of AHSCT in MS and its role compared to novel agents.

## 1. Introduction

Autologous hematopoietic stem cell transplantation (AHSCT) is established as a standard of care for diseases ranging from hematological malignancies to other neoplastic disorders and severe immunological deficiencies [1]. Although autoimmune diseases are characterized by heterogeneous clinical phenotypes, their common characteristic is the development of resistant and rapidly progressing entities in immediate need of intensive management. Novel biological treatments advance at an immense pace but do not reverse organ damage, disability, quality of life, or even life expectancy.

During the last 22 years, severe autoimmune diseases have been treated with heavy immunosuppression and AHSCT aiming to introduce fundamental immunological changes into the structure of the immune system and the function of such naïve lymphocytes which do not promote autoimmunity events. In 1997, our group at G. Papanicolaou Hospital published the preliminary results of phase 1 and 2 pioneering studies in multiple sclerosis (MS) treated with autologous HCT [2]. Since then, great experience has been accumulated in terms of risks, benefits, and economic rates of AHSCT as compared to biological agents. The results of both retrospective and prospective studies became the basis for randomized phase 2 and phase 3 clinical trials in multiple sclerosis (MS), scleroderma, and Crohn's disease. More than 3,000 patients have undergone AHSCT for severe autoimmune diseases worldwide as documented in the European and international registries [3].

Our review is aimed at summarizing state-of-the-art knowledge on basic principles and results of AHSCT in MS and its role compared to novel agents and novel cell-based therapies. A search for original articles was performed in Medline and PubMed with the search terms “multiple sclerosis”, “hematopoietic stem cell transplantation”, “relapsing-remitting”, and “clinical trial” used alone or in combination. Articles were evaluated and included in this review based on their relevance and originality.

## 2. Historical Perspective

In April 1995, our group performed the first AHSCT in patients with progressive MS. Two years later, the initial results of a pilot study on 15 patients with progressive MS were published [2]. In the following years, several centers reported their experience in treating progressive MS patients with AHSCT [4–12]. The multicenter study on behalf of the European Group for Blood and Marrow Transplantation (EBMT) suggested positive early results in the management of progressive MS by AHSCT [13]. Long-term follow-up studies demonstrated a progression-free survival rate ranging from 44% for patients with active CNS (central nervous system) disease to 10% in those without [14, 15]. In addition, accumulating evidence pointed towards significant improvements in disability status and MRI (magnetic resonance imaging) lesions in patients with relapsing-remitting MS who failed to respond to treatment with interferon beta [16–18].

On the basis of the above, consensus recommendations were published in 2012 proposing AHSCT as a therapeutic modality for severe autoimmune diseases at second line or beyond for the treatment of severe deteriorating MS despite standard therapy [3]. These recommendations focused on MS patients in the relapsing-remitting phase with high inflammatory clinical and imaging activities who are rapidly deteriorating despite the use of one or more lines of approved treatments, as ideal candidates for AHSCT. Suitable candidates were also considered patients with malignant (Marburg type) MS and severe disability during the previous year. Clinical relapses or gadolinium-enhancing lesions and/or new T2 MRI lesions on two subsequent scans were considered criteria of inflammatory activity in secondary progressive patients. Patients with these criteria and sustained, clinically relevant increase in disability during the previous year were also recommended as candidates of AHSCT. Ineligibility criteria included the loss of walking ability, except for malignant (Marburg type) forms. All these recommendations were classified as level II recommendations [3, 19].

Since the latest recommendations of 2012, renewed interest in the field has been provided by small-scale case series, case studies, multicenter studies, and meta-analyses in patients with MS responding favorably to AHSCT [20–24]. Outcomes of approximately 800 MS patients have been reported post-AHSCT raising significant issues regarding appropriate patient selection, improvements in AHSCT procedure, and timing of this treatment modality [25]. As a result, the American Society for Blood and Bone Marrow Transplantation (ASBMT) Task Force recommends AHSCT as “standard of care, clinical evidence available,” for patients with relapsing forms of MS (relapsing-remitting or progressive MS with superimposed activity) with high risk of future disability.  Table 1 summarizes current knowledge on ideal MS patients that are candidates for AHSCT.

## 3. Basic Principles of AHSCT in MS

MS is characterized by neuroinflammatory and neurodegenerative components running in parallel. Therefore, AHSCT utilizes conventional immunoablation followed by reconstitution of the immune system to cause immunosuppression and immunomodulation. These effects aim at resetting the immune system [26, 27]. A common misunderstanding in the use of AHSCT in MS treatment is that hematopoietic stem cells represent the therapeutic product. On the contrary, hematopoietic stem cells infused in AHSCT do not differentiate to neurons or oligodendrocytes. Therefore, they cannot repair neurological damage but only provide means to overcome cytopenias and toxicity caused by the immunosuppressive conditioning regimen administered before the infusion of the graft [28, 29].

In addition, AHSCT should be considered a treatment modality in MS rather than one single treatment. Several hypotheses have been proposed in an effort to address the effects of AHSCT on MS patients, concluding that AHSCT may act not only as immunosuppressive but also as immunomodulatory therapy [28, 29]. Several mechanisms through which immune reconstitution therapies such as AHSCT exert their effects have been postulated. These involve the development of a novel immune system lacking pathogenic immune cells [30].

During the advances of the AHSCT procedure, different combinations of immunosuppressive conditioning regimens have been proposed and studied in MS in order to avoid transplant-related toxicities. A basic discrimination classifies conditioning regimens into high, intermediate, and low intensity regimens. High intensity regimens include total body irradiation, cyclophosphamide and antithymocyte globulin (ATG), busulfan or cyclophosphamide, and ATG. ATG at 33% of the dose commonly used to treat aplastic anemia has been administered along with soluble methylprednisolone (0.5 g/day), post-stem cell infusion for in vivo T cell depletion. In some studies, additional ex vivo lymphocyte depletion has been performed using the CD34 cell selection method. Low intensity regimens include cyclophosphamide alone, melphalan alone, or fludarabine-based regimens, while intermediate intensity regimens, such as the BEAM regimen, have been widely incorporated mimicking the current practice in lymphomas [31, 32]. The BEAM regimen, which was initially proposed by our center, is now recommended for MS, consisting of BCNU (300 mg/m^2^) on day -6; etoposide (200 mg/m^2^/d) and Aracytin (200 mg/m^2^/d) on days -5, -4, -3, and -2, and melphalan (140 mg/m^2^) on day -1. Nevertheless, it should be noted that intermediate intensity regimens raise major concerns of long-term toxicity and fertility issues [3]. These issues will be discussed in the following paragraphs.

**Figure 1:**
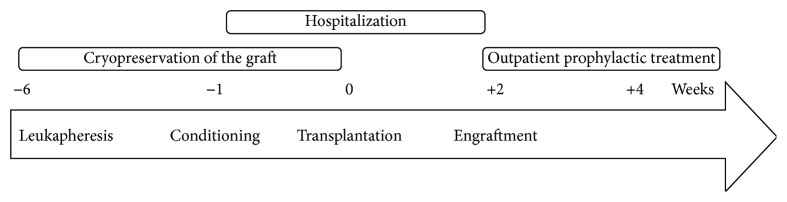
Outline of AHSCT. Key steps of AHSCT include the following. Leukapheresis: mobilization of hematopoietic stem cells following administration of cyclophosphamide and granulocyte-colony stimulating factor (G-CSF). The autologous graft that is harvested from the peripheral blood by leukapheresis is then cryopreserved. Conditioning: a cytotoxic high-dose conditioning regimen is administered during hospitalization for AHSCT. Transplantation: the autologous hematopoietic graft is then reinfused (transplantation), and supportive care is provided during hospitalization for neutropenia until engraftment. Engraftment: following engraftment, close outpatient monitoring and prophylactic treatment are necessary.

Furthermore, there is an evidence-based experience concerning the peripheral blood graft mobilization with cyclophosphamide at 4 g/m^2^ plus growth factor at 10 mg/kg. The hypothesis that growth factor might cause a flare of the original disease has not been confirmed as a major toxicity event during long-term observation [3]. An outline of AHSCT as a treatment modality is presented in Figure 1.

Lastly, it should be noted that transplantation conditions must comply with the international guidelines, which require isolation during hospitalization and intensive prophylactic treatment against infections. Potentially dangerous infections are prevented by the prophylactic use of antibacterial, antiviral, and antifungal treatment for at least 3 months posttransplant. Patient selection is also a critical point for the transplant's success and minimization of treatment-related mortality (TRM). Alternative approaches of outpatient AHSCT have also been recently investigated in MS patients and may be further evaluated in the future [33]. Interestingly, however, a recent report of the EBMT Autoimmune Diseases Working Party showed an association of better progression-free survival with experience, learning, and Joint Accreditation Committee of the International Society for Cellular Therapy and European Society for Blood and Marrow Transplantation accreditation status [34]. In total, these findings suggest that this treatment modality should be considered only in specialized expert and Joint Accreditation Committee of International Society for Cellular Therapy/EBMT-accredited centers.

## 4. Advantages of AHSCT in MS

Classically, MS is classified into relapsing-remitting (RRMS) and progressive forms, including secondary progressive (SPMS), when preceded by RRMS, or primary progressive (PPMS), when progressive from the disease onset [28]. A major pathophysiological difference between relapsing and progressive forms relates to the type of autoimmune response. Relapsing forms are characterized primarily by adaptive responses, while progressive forms by diffuse innate immune response within the CNS and neurodegenerative mechanisms triggered by uncontrolled chronic neuroinflammation [35]. This observation may be important in AHSCT, since this treatment modality mainly targets the inflammatory part of the disease and therefore is considered beneficial in aggressive, treatment refractory RRMS, and active progressive MS with clinical and/or radiological evidence of inflammation.

### 4.1. Efficacy

The most recent systematic reports of efficacy have been provided by two meta-analyses of 15 and 18 studies, respectively [25, 36]. The first meta-analysis included 764 patients with advanced disease (median EDSS (Expanded Disability Status Scale) score of 5.6), the majority of which suffered from progressive MS [25]. In this difficult-to-treat sample, progression post-AHSCT was 17.1% at 2 years and 23.3% at 5 years. Patients with RRMS presented a significantly lower 2-year progression rate [25]. Another important outcome reported in the recent studies is the NEDA (no evidence of disease activity) status (i.e., absence of relapses, progression, and new signs of disease activity on MRI scans). In the recent meta-analysis, the percentage of NEDA patients at 2 years reached 83% (70–92%) and at 5 years, 67% (59–70%) [25]. For a life-long disorder like MS, the long-term outcomes of any therapeutic intervention are particularly relevant. A recent multicenter study explored this issue in 281 patients from 13 countries treated with AHSCT between 1995 and 2006 and followed up for an average of 6.6 years (range 0.2-16 years). The majority of patients (77%) suffered from progressive MS. 5-year progression-free survival was 46%, and overall survival 93%. Neurological progression after AHSCT was associated with older age, progressive instead of relapsing MS, and more than 2 previous disease-modifying therapies [37]. The second meta-analysis included 732 patients showing a progression-free survival (PFS) of 80% in patients transplanted with low and intermediate intensity regimens. In addition, patients with RRMS showed a PFS of 85% [36].

Another important aspect of efficacy is reflected in improvements of quality of life. AHSCT resulted in improved quality of life in two studies [20, 38]. Improvements in quality of life following AHSCT may be also associated with improvements in fatigue, since a recent study has provided relevant evidence in aggressive MS [39].

### 4.2. Cost of Treatment

Despite the lack of data on direct comparisons between AHSCT and other treatment modalities, indirect comparison of costs seems to favor AHSCT. To be more specific, Hartung and colleagues calculated the annual cost of MS treatment with immunosuppressive or immunomodulatory drugs at approximately 50,000–70,000 USD in 2015 [40]. This cost accrues indefinitely in contrast to the cost of AHSCT which is a one-time treatment not expected to cause direct costs posttransplant. The median cost of AHSCT with high intensity regimens has been calculated at approximately 140,000 USD in 2017 [41].

## 5. Disadvantages of AHSCT in MS

### 5.1. Safety and Toxicity

In the abovementioned meta-analyses, treatment-related mortality (TRM) was 2.1% and 1.34%, respectively [25, 36]. TRM was higher in studies with a lower proportion of patients with RRMS, as well as patients with higher baseline EDSS [25]. There is a general consensus that TRM is lower in newer studies (less than 1%) [42].

Nevertheless, AHSCT confers acute and late toxicities that are rather limited compared to allogeneic transplantation and are similar to chemotherapy-induced toxicities. Acute toxicities such as alopecia, infections, mucositis, and gastrointestinal symptoms are addressed by proper supportive care [43]. Late toxicities are of multisystem nature, involving the endocrine system, autoimmune phenomena, and infertility. Proper counseling and monitoring by both transplanters and neurologists are required according to current guidelines [44]. Upcoming multicenter randomized controlled studies are expected to provide further high quality data on the role of AHSCT in MS patients [45].

### 5.2. Other Factors

Despite encouraging results in efficacy, safety, toxicity, and economic cost, several factors not associated with scientific validity limit the broad application of AHSCT. These include both factors associated with transplant units that may have limited resources to treat these patients, treating neurologists that may not be familiar with this procedure, and healthcare reimbursement depending on the healthcare system [45]. Overcoming these obstacles is needed to offer AHSCT in selected patients according to state-of-the-art treatment recommendations.

## 6. AHSCT and Pediatric MS

Although pediatric MS is generally benign in the short term, it may progress to severe forms of disease. Of note, particular forms of pediatric MS severely affect brain development. In this context, the approach of AHSCT seems appealing in children with MS based on the rationale of a one-time treatment that promises elimination of inflammation. These conditions are expected to allow normal brain development avoiding long-term exposure to immunomodulatory or immunosuppressive agents and improvement of quality of life for a long period of time. Low intensity conditioning regimens might be preferable in the pediatric setting aiming to limit long-term toxic effects of cytotoxic agents. In this context, increased awareness from transplanters and neurologists is warranted to carefully monitor late effects of transplantation according to current recommendations [44, 46].

The first report of treatment with AHSCT in children with MS was recently published in a registry-based study of the Autoimmune Diseases Working Party and Pediatric Diseases Working Party of the EBMT [47]. This multicenter study reported outcomes in 22 patients. Mobilization of peripheral blood stem cells was achieved with the standard method of cyclophosphamide and growth factor administration. The majority of patients (13 out of 22) received a low intensity conditioning with cyclophosphamide 200 mg/kg, whereas 9 out of 22 received an intermediate intensity conditioning with BEAM. Regarding safety, only one patient experienced unexpected serious adverse events. In terms of efficacy, 100% progression-free survival was achieved post-AHSCT, with no patient deteriorating from the baseline. Improvement in EDSS was observed in 76% of patients. Taken together, these results suggest that AHSCT is an adequate treatment for pediatric-onset MS, based on the International Pediatric Multiple Sclerosis Study Group guidelines for assessment of treatment efficacy [48]. Although no data exist on a direct comparison of AHSCT with other treatment modalities in pediatric MS, indirect comparisons with natalizumab and rituximab treatment are encouraging for AHSCT outcomes [49, 50].

## 7. AHSCT and Novel Agents in MS

Over the last 25 years, the MS treatment pipeline has dramatically changed. Several novel treatment options as well as high-efficacy therapeutic drugs have emerged including glatiramer acetate, mitoxantrone, natalizumab, fingolimod, teriflunomide, dimethyl fumarate, alemtuzumab, daclizumab, cladribine, and ocrelizumab [51, 52]. In general, the majority of these immunomodulatory or immunosuppressive drugs need to be administered continuously in order to control disease activity. This “maintenance” approach comes along with several limitations: complications, health cost, and patient's adherence to a life-long treatment. Importantly, despite the expanding therapeutic options, a portion of patients responds insufficiently, whereas others present contraindications or complications to immunomodulatory or immunosuppressive drugs requiring an alternative therapeutic approach [53]. The most recent position paper by the ASBMT does not comment on the role of novel agents [54]. Therefore, updated recommendations regarding the role of AHSCT in the therapeutic algorithm of MS are required in the era of novel agents. Existing guidelines place AHSCT as rescue treatment after failure of second-line treatment along with alemtuzumab and other off-line treatments (ocrelizumab, rituximab) [55]. In addition, the Belgian group most recently recommended AHSCT in aggressive RRMS patients after treatment failure of at least one highly effective treatment (2 courses of alemtuzumab or at least 6 months of treatment with mitoxantrone, cyclophosphamide, natalizumab, rituximab, and ocrelizumab). The same group recommended AHSCT in progressive patients with active disease only in case of ocrelizumab treatment failure, since ocrelizumab is indicated for primary progressive patients [56].

The critical question whether AHSCT might be used in combination with immunomodulatory or immunosuppressive drugs cannot be answered on the basis of existing data. The only available evidence stems from a recent study in patients that underwent AHSCT following discontinuation of natalizumab. A minimum period of 6 months from the last natalizumab infusion was adopted with the use of a bridging therapy (cyclophosphamide or corticosteroid methylprednisolone). AHSCT was performed with acceptable toxicity with no fatalities or serious complications such as progressive multifocal leukoencephalopathy (PML). Disease reactivation in the patients who received AHSCT was observed only during wash-out/bridging therapy whereas following AHSCT, all cases were free from disease activity period [57].

## 8. Novel Cell-Based Therapies in MS

**Figure 2:**
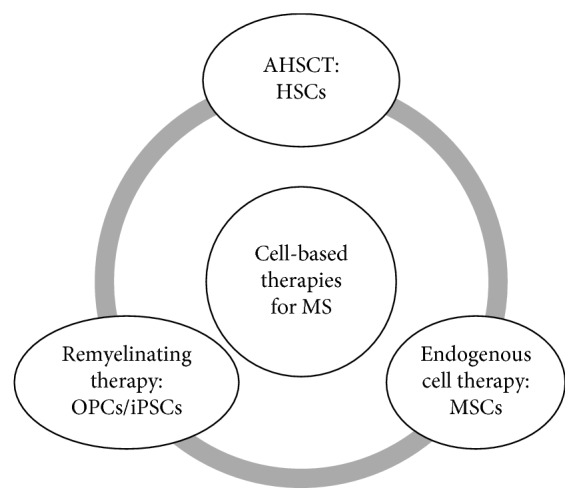
A schematic representation of cell-based therapies in multiple sclerosis. AHSCT: autologous hematopoietic stem cell transplantation; MSCs: mesenchymal stem cells; OPCs: oligodendrocyte progenitor cells; iPSCs: induced pluripotent stem cells.

In contrast to AHSCT, the cells are the therapeutic product in novel cell-based therapies that are currently under investigation in MS [58]. These are classified into two major categories: endogenous cell therapy including mesenchymal stem cells (MSCs) and cell-based remyelinating therapy including oligodendrocyte progenitor cells (OPCs) and induced pluripotent stem cells (iPSCs) [59].  Figure 2 summarizes cell-based therapies in MS. Except for endogenous problems that need to be addressed in further studies, ethical considerations represent an important aspect of research in cell-based therapies. Studies with cell-based therapies need to strictly comply with recent guidelines for human embryonic stem cell research [60].

### 8.1. Endogenous Cell Therapy

Although early studies have suggested that MSCs differentiate into both neurons and oligodendrocytes [61], potential repair-promoting actions of MSCs in the CNS are based rather on their paracrine mechanisms of action than the phenomenon of transdifferentiation. Therefore, the potential use of MSCs in MS in the context of endogenous cell therapy would be through amelioration of different pathological processes that contribute to tissue damage [62]. In line with this hypothesis, MSCs from MS patients have demonstrated similar growth in culture, differentiation potential, surface antigen expression, and immunomodulatory properties with MSCs from non-MS individuals [63–65]. Nevertheless, other studies have shown functional differences of MSCs [66, 67].

Clinical reports of applications of cell-based therapies in patients with different underlying diseases have shown serious clinical complications including transient aseptic meningitis [68], acute disseminated encephalomyelitis [69], glioproliferative spinal cord tumor [70], and severe visual loss [71]. A number of additional issues remain to be resolved including the appropriate cell dose, number of infusions, and type of cell preparation, as well as cryopreservation, donor variance, culture expansion, immunogenicity, epigenetic reprogramming, and senescence [72, 73]. Therefore, further clinical trials are warranted in this experimental field. Larger phase 2 studies of bone marrow-derived cells [74, 75] and culture-expanded MSCs [76] are ongoing.

### 8.2. Remyelinating Therapy

Remyelinating therapy by OPCs has been of particular interest in progressive MS. However, OPCs' use in clinical applications is limited by a number of issues. First, direct injection of OPCs into the CNS is necessary, since OPCs do not have the capacity to traffic from blood or cerebrospinal fluid into the CNS but do migrate within the CNS [77]. Second, OPCs are retrieved in limited numbers from fetal tissue and have limited proliferative capacity when cultured. Alternative approaches to overcome this problem would require the use of allogeneic cells in immunosuppressed recipients or the generation of OPCs from autologous iPSCs. Another alternative approach would be to find agents acting on intrinsic OPCs to stimulate remyelination. Third, OPCs are already present in chronic lesions of MS, indicating that remyelination is rather limited by other factors, such as their interaction with the axons and the microenvironment [78]. As a result, administration of OPCs may not be successful in MS patients. Therefore, proof-of-principle studies are needed in MS patients to provide safety and feasibility data on remyelination therapy.

Lastly, iPSCs have the potential to differentiate to OPCs and could therefore represent a suitable source of autologous cell-based therapy. Recent studies of iPSC-derived neural precursors have observed neuroprotective but not remyelinating properties [79]. In addition, preclinical models have shown encouraging results in MS [80, 81]. However, earlier applications of iPSCs have raised concerns on malignant transformation or immune rejection [82, 83]. Future studies remain to further characterize the potential benefit of iPSCs in MS.

## 9. Conclusions and Future Perspectives

In this review, we have summarized accumulating experience on the use of AHSCT in MS patients. Over the years, safety and toxicity of AHSCT have improved along with improved efficacy in selected patient populations. As a result, consistent data point toward a high percentage of “no evidence of disease activity,” especially in RRMS patients. Although no direct comparisons are available, these results compare favorably with conventional treatments paving the way for the use of AHSCT in carefully selected MS patients even in the era of multiple novel treatment options. Beyond AHSCT, other cell-based therapies are currently investigated in MS patients with promising results. However, there is no definitive evidence for efficacy in MS, and novel cell-based therapies should be considered only in the context of rigorous clinical trials [59]. Therefore, future studies need to further explore combinations of cell-based therapy with conventional treatment in an effort to improve outcomes of MS patients.

## Figures and Tables

**Table 1:** Characteristics of MS patient candidates for AHSCT.

Characteristics of MS patient candidates of AHSCT	Level of evidence [19]
Relapsing-remitting MS [84]	B-R
2 or more clinical relapses or 1 relapse and MRI gadolinium-enhancing lesion(s) at a separate time within the previous 12 months despite receiving treatment with DMT [84]	B-R
EDSS 2.0-6.0 [84]	B-R
Younger patients with shorter disease duration [85, 86]	B-NR
Malignant (Marburg type) MS and severe disability [85]	B-NR
No comorbidities [20, 85]	B-NR
Able to ambulate independently [3]	B-NR

AHSCT: autologous hematopoietic stem cell transplantation; MS: multiple sclerosis; DMT: disease-modifying therapies; EDSS: Expanded Disability Status Scale; B-R (randomized): moderate-quality evidence from 1 or more randomized clinical trials; B-NR (nonrandomized): moderate-quality evidence from 1 or more well-designed, well-executed nonrandomized studies, observational studies, or registry studies.
